# Annotations

**Published:** 1902-11-22

**Authors:** 


### Annotations.

### The London County Council and Overcrowding.

The London County Council has shown, we are glad to note, an increasing interest in the question of the housing of the poor, the idea mostly favoured being that which entails the removal to the suburbs of thousands of residents in the more congested districts. Such a plan would probably prove highly beneficial to the wives and families of the artisan class, but it entails a greater strain upon the artisan himself unless he is fortunate enough to be able to obtain work at a place readily accessible from the district in which he resides. In any case the distances to be traversed by the heads of families can never be inconsiderable in a great city like London, and they must mean very early rising and extra strain for the worker himself. Still the population as a whole is likely to benefit from the adoption of this system, providing care is taken to secure an adequate number of suitable dwellings within the districts where certain trades are carried on, for they render it imperative that the employee shall be always accessible to the employer at short notice. Our meaning is, that in the case of tailors, for instance, it is essential that the worker shall reside within a short distance of the shop which supplies him with work, whether his master provides the workroom, or he has to take his work out to do at home. Londoners are anxious to be assured that the County Council fully recognises this essential exception to any scheme for the removal of large masses of the poorer population of London from the centre to the suburbs, where sites are less expensive, air and light more abundant, and the conditions more favourable to a healthy existence for the families of the artisan class. We hope the London County Council will promptly give this assurance to the public.

### Restaurant Kitchens.

As a very considerable proportion of the population of London eats at least one meal a day in restaurants the investigations which several Medical Officers of Health have recently been making regarding restaurant kitchens are matters of widespread interest. It is only lately that it has been possible to bring these kitchens under medical inspection, but the Secretary of State has now decided that they are under the operation of the Public Health Act, not so much because customers' food is prepared in them as because cooks and scullions work in them. In the City of London, Dr. Collingridge reports that there are 279 restaurant kitchens, of which 211 are underground. In these work 553 men and 3,041 women. Inspections have been made also in other districts, but these are the most important. It is to be regretted that the conditions of these kitchens left in many cases much to be desired. Many were dirty, some were ill-ventilated, and in some sanitary conveniences were either lacking or defective. Dr. Dudfield noted that as a rule the workmen's dining-rooms had cleaner kitchens than those of higher pretensions, which is good news for the working men, but not for other people. Perhaps the fact that there is less variety of food to be prepared in them makes cleanliness easier. Moreover, in these restaurants the demand on the attention of cooks and kitchenmaids is not so continuous. The business comes with a rush, but it is soon over, and there is more leisure to clear away before the next influx than in places where luncheon goes on almost until it is time for tea, and the demand for that does not cease until the preparations for dinner are in full swing. This, however, although an explanation, is no excuse for any lack of cleanliness in higher-priced restaurants. The charges made in them are surely sufficient to make it possible for the proprietors to engage a sufficient staff to keep their kitchens clean, and also to pay for the necessary periodic renovations. Now that medical officers have the right to enter and report on restaurant kitchens, we may hope that public opinion will support them in raising the standard of cleanliness therein. Proprietors know how the exposure of a dirty kitchen would affect their trade.

### The Metropolitan Asylums Board.

The annual report of the Statistical Committee of the Metropolitan Asylums Board, which was presented last Saturday, shows, among other things, how great is the work involved in providing for the isolation and treatment of those infectious diseases which fall to the care of that important public body. During the year 1901 the cases of scarlet fever which were admitted to the hospitals of the Board numbered 14,539. There were 7,622 cases of diphtheria, 1,129 of enteric fever, 13 of typhus fever, and 2,365 of "other diseases," many of which were cases the nature of which had been erroneously diagnosed by the practitioners in attendance, and under whose certificates they had been received. The total number admitted was the highest yet reached in any one year. Average death-rates over large numbers are always of interest as an index of the intensity or otherwise of the current type of disease, and here we find that the death rates were among scarlet fever cases 3·81 per cent., diphtheria cases 11 15 per cent., enteric fever cases 14 22, and typhus fever cases 30·77. The diphtheria death-rate was the lowest yet noted in the records of the Board, while it is worthy of note that rare as typhus fever has become it retains its old pre-eminence as one of the most fatal of the fevers. Of small-pox, 1,743 cases were admitted and 257 died. So far as the Metropolitan Asylums Board is concerned, it is stated that the cost of the late outbreak may be taken at the round figure of £500,000, which, having regard to the area and population dealt with, is not considered a large amount. If the whole cost were paid at once it would be defrayed by a 3d. rate, whereas it has not infrequently happened that a small provincial town has paid more heavily for a single visitation of small pox. Even districts just outside the borders of London have, in the recent epidemic, been much more severely mulcted. The committee point out, with some satisfaction, that in the Board's fever hospitals, including the Southern Convalescent Hospital now in course of erection, the accommodation provided will shortly be three times what it was 10 years ago. The prevalence of diphtheria, however, and the popularity of the hospitals causes the demand for beds to tread upon the heels of the supply.

